# Supplementary material for: Comparison of heart rate obtained from shorter duration Holter recordings to 24-hour mean heart rate in dogs with atrial fibrillation
Source: PLoS One. 2020 Nov 2;15(11):e0241620. doi: 10.1371/journal.pone.0241620 (PMC7605697; doi:10.1371/journal.pone.0241620)
Supplement: Attachment — Submitted filename: Response to Reviewers.docx [file pone.0241620.s001.docx]

PONE-D-20-23023
Comparison of heart rate obtained from shorter duration Holter recordings to 24-hour mean heart rate in dogs with atrial fibrillation
PLOS ONE

Dear Dr. Sanders,

Thank you for submitting your manuscript to PLOS ONE. After careful consideration, we feel that it has merit but does not fully meet PLOS ONE’s publication criteria as it currently stands. Therefore, we invite you to submit a revised version of the manuscript that addresses the points raised during the review process.

The Authors should respond to all of the Reviewers' comments. One of the major comments of the Reviewers was the potential added benefit of shorter time periods for Holter monitoring over 24 hour monitoring that usually is carried out, due to the fact that animals would still have to undergo the procedures for placing the Holter. Therefore this, especially, would need to be addressed in a revised manuscript. Furthermore, the Reviewers also noted that the statistical analysis of the manuscript should be strengthened.

Please submit your revised manuscript by Sep 21 2020 11:59PM. If you will need more time than this to complete your revisions, please reply to this message or contact the journal office at [plosone@plos.org](mailto:plosone@plos.org). When you're ready to submit your revision, log on to <https://www.editorialmanager.com/pone/> and select the 'Submissions Needing Revision' folder to locate your manuscript file.

Please include the following items when submitting your revised manuscript:

- A rebuttal letter that responds to each point raised by the academic editor and reviewer(s). You should upload this letter as a separate file labeled 'Response to Reviewers'.
- A marked-up copy of your manuscript that highlights changes made to the original version. You should upload this as a separate file labeled 'Revised Manuscript with Track Changes'.
- An unmarked version of your revised paper without tracked changes. You should upload this as a separate file labeled 'Manuscript'.

If you would like to make changes to your financial disclosure, please include your updated statement in your cover letter. Guidelines for resubmitting your figure files are available below the reviewer comments at the end of this letter.

If applicable, we recommend that you deposit your laboratory protocols in protocols.io to enhance the reproducibility of your results. Protocols.io assigns your protocol its own identifier (DOI) so that it can be cited independently in the future. For instructions see: [http://journals.plos.org/plosone/s/submission-guidelines#loc-laboratory-protocols](https://urldefense.com/v3/__http:/journals.plos.org/plosone/s/submission-guidelines*loc-laboratory-protocols__;Iw!!HXCxUKc!grLQiqQkx2ZabI8a2qbYpJAeolBPWgHO9aglqUEeiX41sgCJpXtgY3Mm5Bo$)

We look forward to receiving your revised manuscript.

Kind regards,

Daniel M. Johnson, PhD
Academic Editor
PLOS ONE

Journal Requirements:

When submitting your revision, we need you to address these additional requirements.

1. Please ensure that your manuscript meets PLOS ONE's style requirements, including those for file naming. The PLOS ONE style templates can be found at

<https://journals.plos.org/plosone/s/file?id=wjVg/PLOSOne_formatting_sample_main_body.pdf> and

<https://journals.plos.org/plosone/s/file?id=ba62/PLOSOne_formatting_sample_title_authors_affiliations.pdf>

2. We note that you have stated that you will provide repository information for your data at acceptance. Should your manuscript be accepted for publication, we will hold it until you provide the relevant accession numbers or DOIs necessary to access your data. If you wish to make changes to your Data Availability statement, please describe these changes in your cover letter and we will update your Data Availability statement to reflect the information you provide.

Please find the DOI for the data.

Gunasekaran, Tamilselvam (2020), “Atrial fibrillation dogs”, Mendeley Data, v1http://dx.doi.org/10.17632/hbwy6bn48n.1

3. PLOS requires an ORCID iD for the corresponding author in Editorial Manager on papers submitted after December 6th, 2016. Please ensure that you have an ORCID iD and that it is validated in Editorial Manager. To do this, go to ‘Update my Information’ (in the upper left-hand corner of the main menu), and click on the Fetch/Validate link next to the ORCID field. This will take you to the ORCID site and allow you to create a new iD or authenticate a pre-existing iD in Editorial Manager. Please see the following video for instructions on linking an ORCID iD to your Editorial Manager account: <https://www.youtube.com/watch?v=_xcclfuvtxQ>
[Note: HTML markup is below. Please do not edit.]

Reviewers' comments:

Reviewer's Responses to Questions

**Comments to the Author**

1. Is the manuscript technically sound, and do the data support the conclusions?

The manuscript must describe a technically sound piece of scientific research with data that supports the conclusions. Experiments must have been conducted rigorously, with appropriate controls, replication, and sample sizes. The conclusions must be drawn appropriately based on the data presented.

Reviewer #1: Yes

Reviewer #2: Yes

Reviewer #3: Yes

2. Has the statistical analysis been performed appropriately and rigorously?

Reviewer #1: No

Reviewer #2: Yes

Reviewer #3: I Don't Know

3. Have the authors made all data underlying the findings in their manuscript fully available?

The [PLOS Data policy](https://urldefense.com/v3/__http:/www.plosone.org/static/policies.action*sharing__;Iw!!HXCxUKc!grLQiqQkx2ZabI8a2qbYpJAeolBPWgHO9aglqUEeiX41sgCJpXtgUk1Sm7g$) requires authors to make all data underlying the findings described in their manuscript fully available without restriction, with rare exception (please refer to the Data Availability Statement in the manuscript PDF file). The data should be provided as part of the manuscript or its supporting information, or deposited to a public repository. For example, in addition to summary statistics, the data points behind means, medians and variance measures should be available. If there are restrictions on publicly sharing data—e.g. participant privacy or use of data from a third party—those must be specified.

Reviewer #1: Yes

Reviewer #2: Yes

Reviewer #3: Yes

4. Is the manuscript presented in an intelligible fashion and written in standard English?

PLOS ONE does not copyedit accepted manuscripts, so the language in submitted articles must be clear, correct, and unambiguous. Any typographical or grammatical errors should be corrected at revision, so please note any specific errors here.

Reviewer #1: Yes

Reviewer #2: Yes

Reviewer #3: Yes

5. Review Comments to the Author

Please use the space provided to explain your answers to the questions above. You may also include additional comments for the author, including concerns about dual publication, research ethics, or publication ethics. (Please upload your review as an attachment if it exceeds 20,000 characters)

Reviewer #1: The authors provide a study that examined whether periods substantially shorter than 24hours of Holter monitoring (ambulatory ECG monitoring) could reasonably accurately reflect the 24 hour average heart rate in dogs with atrial fibrillation.

I have several concerns about the analyses.
First, in previous studies of mean heart rate, investigators suggested trimming the Holter recordings to exclude the first 3 hours and final hour of the recording (<https://pubmed.ncbi.nlm.nih.gov/19645836/>, see figure 2B) as this removes the effect of stress and anxiety experienced by most dogs when applying the Holter and when returning to have the Holter removed. The residual 20 hours more accurately reflect the true “at home” average heart rate. The authors should consider this approach to see how this affects their interpretation of agreement. They would obviously remove the short periods from these peripheral time periods (because these, too, would be affected by anxiety etc).

We did undertake such a measure to remove the initial portion of the Holter recording that is likely affected by the stress of a hospital visit. We removed the initial portion (> 3 hrs in every case) of the Holter recording from the time of Holter application to midnight on the day of Holter application. Therefore, for each dog 24 hours of Holter data starting at midnight to midnight next day was extracted for further analysis. We noted this in 136-137 lines. We have added additional lines (137-141) to the manuscript to explain this process in detail.

The statistical analysis section needs expanding. This reviewer cannot determine what analyses were applied for what purposes. For example, what “study variables” did the authors examine by Spearman Rank Correlation?

We have expanded on the statistics section. However, per reviewer 2 recommendations we have removed some portions of the statistical analysis performed.

The accuracy of short periods might be very dependent on specific dogs. Did the authors account for dog in their analysis? In other words agreement might be high for some dogs and low for others. Examining the % agreement by dog might reveal such a “between dog” effect.

We agree that % agreement to 24 hours mean HR is dog dependent. We have now added an additional summary figure (new fig 1) that highlights this information.

A further approach the authors should consider is to randomly select a 5-minute, 30-minute, 1 hour, 2-hour and 3-hour segment from each dog and see how often these randomly selected segments agree within the predefined limits. This would represent the clinical reality of a client obtaining a heart rate at home.

We did think about performing a random selection approach as recommended by the reviewer. One of the problems with such an approach is that a random selection of a short duration ECG recordings will result in inclusion of sampling periods that are not uniform among all dogs. For example, in one dog the sampling may occur during periods of rest while in others sampling may occur during extreme activity. We believe this would limit the usefulness of the direct clinical application of a shorter duration sampling. The authors believe that a better approach would be if prospective sampling was done under uniform conditions such as at rest or specific time of the day, as it would directly translate to clinical application. Unfortunately, given the retrospective nature of our study with incomplete Holter diary entries, we could not perform analysis limited to specific situations.

Minor comments:
Line 88-90. The authors state the limitations of Holters (cost, duration, compliance). However, the study examines Holter recordings and does not provide or discuss what alternatives to Holter monitoring exist or that could substitute Holter monitoring (and this reviewer knows of no such alternatives). Therefore, this statement seems rather contrived and without a solution.

Thank you for noting this. We understand how our statement is incomplete. We have added additional information clarifying our goal in the introduction and limitation sections.

Lines 177-180. If the average rates are not different statistically, then one group cannot have a “slightly higher” rate than the other. Also, a P value should be provided for all statistical testing, whether significant or not. Please amend.

Requested changes made.

Line 177-180. The average HR for dogs with AF that were not being administered any rate control therapy seem considerably lower than previously documented in dogs with secondary AF. Do the authors have any explanation for this rather low rate? (most previous studies suggest rates exceeding 160-190 bpm). The rates for the group under rate control therapy appears to be consistent with previous observations.

We did notice such a discrepancy from previous studies. There are several reasons for the lower HR in the untreated group in this study. The data was edited to remove the initial portion of the Holter recording which is different compared to previous studies (only first 3 hours in the previous studies). To our knowledge, there is only one study where the 24-hour Holter mean HR was reported for the untreated group in the past (Gelzer AM, etal. Combination Therapy with Digoxin and Diltiazem Controls Ventricular Rate in Chronic Atrial Fibrillation in Dogs Better than Digoxin or Diltiazem Monotherapy: A Randomized Crossover Study in 18 Dogs). In the Gelzer study only dogs above a 24-mean HR of 140 bpm were included. Therefore, the true mean 24-hour HR in a group of untreated dogs is unknown. Additionally, in our institution Holter monitoring is routinely performed in most dogs to assess the need for rate control therapy prior to initiation of any rate control therapy. Similar to previous observations, we have noted in some of these dogs with extremely high in-hospital ECG HR that the at home 24-hour mean HR is low and does not warrant any rate control therapy. Therefore, portion of our untreated group is formed by such patients with low 24hour mean HR at home. We have added a few lines in the limitation’s sections to clarify this.

Line 185-186. Could the authors provide scatter plots for each of the short duration recordings vs the Holter recordings plotted as average heart rates? Although the P value for their observation is “not significant”, with only 10 dogs in each group, the probability of this being a false negative is quite large.

We have removed the correlation analysis per reviewer 2 recommendations. From what we understand the reviewer is asking to provide scatter plot for pairs of each shorter duration ECG recording HR to 24 hours mean HR? It would be cumbersome to do. For example, for 5 min recordings there is 288 separate 5 min vs 24-hour plots needs to be done for each dog. Unless a specific 5 min sample is selected (the limitations of which we addressed in the previous comment) the utility of such a plot is limited. If the reviewer is asking for average of all 5 min HRs for each dog vs 24 hour mean HR scatter plot, then we believe that the average of all 5 min would be essentially same as the 24 hours mean HR.

Line 204-213. The authors suggest that a 5-minute ECG recording (in-clinic) would mostly agree to within 20% of the Holter-determined average HR. However, this is NOT what the authors showed. To demonstrate that specific idea, the authors would need to examine the 5 minute intervals within the 1st hour of Holter application (while the dog was still in the clinic and stressed) with the average at-home HR. How many of the 240 5-minute intervals obtained during the first hour came within 20% of the average at-home heart rate? If the authors suggest that clients could obtain a 5-minute ECG at home on their pets, the authors would have to suggest how such a feat would be possible, and also demonstrate that doing this would not alter the level of anxiety in the dog during at-home acquisition.

As the reviewers allude to there are several possible at home ECG monitoring devices that a client can use to obtain short term ECGs (AliveCor, cutaneous applied loop recorder (previously shown at our institution), patch recorders). While devices such as AliveCor requires owner-dog interaction the devices such as Cutaneous loop recorders can be activated to collect ECG with minimal interaction. With ever evolving technology there is likely further advances in ECG technology that would minimize the need for dog interaction thereby anxiety during data collection. We have added additional descriptions in introduction section and limitations section to clarify this.

Line 215-220. Several of the “health collars” (e.g. PetPace) can record ECGs in dogs, at least in sinus rhythm. Whether they can accurately count heart rates in dogs with atrial fibrillation remains to be determined. If they can, then they provide continuous monitoring, or can record variable periods for as long as desired (limited by battery life). Therefore, such devices (similar to the iWatch etc, used in humans) are available, but in limited supply.

Agree that they are available and agree that they are currently untested. We have that research planned.

Reviewer #2: General comments – this manuscript describes a retrospective analysis of subsets of ECG data recorded during a 24 hour Holter recording. While providing interesting preliminary data, I wonder how much less practical difficulty there might be in practice in obtaining shorter Holter recordings compared to a 24 hour recording? The dog still has to have the monitor fitted and wear it for several hours (preferably from the point the dog is relaxed at home, not from the initial time of Holter fitting) – if the benefits are “cost, recording duration and owner compliance” how much difference would shorter recordings truly make? I don’t have a good feel for how much less companies would charge to analyse shorter recordings, unfortunately. The main advantage I can see would be in those cases that the leads became detached after less than 24 hours – if it could be shown that the data was valid if at least 2-3 hours of recording was available once the dog was at home and relaxed that could prevent the need to start all over again. My specific comments, questions and observations are detailed below.

Dear reviewer, our goal with this study was not to replace a longer Holter recording with a shorter Holter recording. Rather we sought to assess the accuracy of a shorter ECG recording with available Holter data as a surrogate to shorter duration ECG devices for HR assessment in dogs with AF. While the data from this study could not be directly extrapolated to shorter duration device obtained HR information (due to factors such as human-dog interaction and such action on HR while collection). We have added additional information in introduction and limitations sections to further clarify this.

Introduction
Line 62 – should read “…remain a concern.”

Recommended changes made

Lines 63-65 – this sentence is identical to lines 59-61. I recommend deleting the sentence in lines 59-61, as it makes most sense to keep this information at the end of the paragraph.

Recommended changes made

Lines 67-79 – you mention that pulse assessment can be used to assess heart rate, but do not discuss later in the paragraph whether this would provide an accurate reflection of heart rate (which seems unlikely, due to pulse deficits, etc.).

Recommended addition made.

Lines 91-93 – how do you propose that the Holter is fitted without “the stress of hospital visits”?

See comments above

Results
Line 151 – just an idle observation, but I’m surprised only 20 dogs met the inclusion criteria over a 7 year period. Were there a lot of recordings that were <24 hours when edited?

Yes, the removal of the initial portion of the Holter recording resulted in less than 24 hours of Holter data in many AF dogs and therefore excluded from the study.

Lines 152-153 and 154-155 – personally, I would just report the range rather than the range and the IQR, as I don’t think the latter adds anything of real value.

Recommended changes made

Lines 177-180 – please don’t describe non-significant differences. This should simply read “There was no significant difference in heart rate between dogs receiving HR control therapy (121+/- 33bpm) and those not receiving HR control therapy (134 +/- 29 bpm).”

Recommended changes made.

Lines 180-182 – Table 1 and Figure 1 essentially show the same information twice. Please remove either the Table or the Figures.

Per recommendation the figure 1 is removed now.

Lines 182-184 – I’m not sure why this correlation is clinically interesting or important – please explain why you performed this test.

Lines 184-186 – given that rate control status had no significant impact on heart rate, why would you imagine that it would have an impact on accuracy? Again, this seems to be a statistical test that isn’t testing a hypothesis.

Excellent points; our hypothesis was that in dogs with higher mean 24-hour HR’s, the accuracy of shorter duration ECG will be better. We anticipated that dogs with higher 24-hour mean HR’s will have limited ability to reach lower HR ranges, and potentially less of a deviation from 24 hour mean HR in one direction resulting in narrower band width of shorter duration HR averages. But you are correct, since the HR was not different between two group the accuracy is unlikely to be different nor there likely be a relationship to the 24 hours mean HR. We have removed these analyses now.

Table 1
The information displayed represents “Median (95% CI)” NOT “(Median +/- CI)” – please edit this accordingly.

Recommended correction made.

Discussion
Lines 206-207 – I don’t think that “agreement” changes – you are just being more lenient about what you are willing to accept as “accurate”.

We have made modifications to this statement.

Lines 209-215 – based on your stated mean heart rate of 126 bpm, a 20% inaccuracy gives a heart rate of 151 bpm, which is likely to be interpreted very differently than a mean of 126 by most clinicians. This is clearly potentially problematic – please expand the discussion to reflect this.

We have added additional statements as recommended.

Lines 215-220 – this is the first time you have mentioned alternative methods of obtaining an ECG recording in the home environment – which potentially changes ho you might be positioning shorter recordings (although I don’t relish the idea of asking a client to hold an AliveCor against their dog for an hour!). Please add this information to the Introduction (with a brief summary of the work that has already been done looking at accuracy of such recordings in the home environment for monitoring of AF).

Recommended description on previous work is now added to the introduction section.

Line 236 – please remove the grocer’s apostrophe (the “s” in ECGs denotes that this is plural rather than denoting possession).

Recommended change made

Lines 226-228 – is it not equally likely that these would consistently underestimate 24 hour averages (simply because they wouldn’t include the periods of exercise and excitement that also influence mean heart rate over 24 hours)? So you might be at risk of thinking that your rate control was better than it actually is?
Lines 237-248 – I am still not sure why you did this test in the first place (it feels like a fishing trip) so my preference would be to remove this from the discussion.
Lines 248-251 – this is possible, of course, but I think it’s very unlikely (see previous comment regarding lines 209-215).

These are great points. Taking your comments into consideration we have removed comments about prospective evaluations performed at resting ECGs.

Lines 255-257 – this would still rely on owners to record these times accurately – is there a way to mitigate this as a potential limitation to future studies?

We have removed this comment now per recommendation above. But in general, the owners would only be asked to record ECGs related to specific activities such as resting, sleeping or activity. With the time stamps from the owner recorded ECGs we can certainly match to a concurrently applied Holter recorder.

Conclusions
Lines 260-262 – surely there is experimental evidence that would help determine guidelines for what is acceptable accuracy, rather than simply relying on the judgement of the clinician?

While there are some evidence on what an acceptable HR for an AF dog is, the authors are unaware of any studies that specifically looked at an acceptable accuracy for a ECG device for measuring HR in dogs with AF. In veterinary patients of AF we believe that the clinician judgement of an accuracy is likely vary and as such we would like to leave the conclusions statements in the current version.

Reviewer #3: The purpose of this study was to determine if the heart rate measured for shorter time periods (5 minutes, one, two and three hours) would equate to the mean rate as determined by 24 hour electrocardiographic monitoring. The mean heart rate for each of these four time intervals was assessed for its ‘closeness’ (within 10%, 15% or 20%) to the 24 hour mean rate. The authors embarked on this investigation with the justification that performing a 24 hour Holter for shorter periods of time would be less problematic for owners and dogs. The conclusion of this paper is that none of the shorter time periods would equate to the mean 24-hour heart rate, but the longer the recording the closer the short surveillance heart rate was to the full recording.

This is a very focused study to determine if short recordings are adequate in the evaluation of rate during atrial fibrillation. The authors state that there are no other methods currently available to determine heart rates in a dog for longer than one hour. However, an Internet search will reveal that individuals are adapting noninvasive monitoring such as Fit Bits for dogs. Additionally, other devices have been developed. (See reference: Yasin Cotur, Michael Kasimatis, Matti Kaisti, Selin Olenik, Charis Georgiou, Firat Güder. Stretchable Composite Acoustic Transducer for Wearable Monitoring of Vital Signs. Adv. Funct. Mater. 2020, 30, 1910288. Doi.org/10.1002/adfm.201910288) These may serve as a way to get minimal information (and likely inadequate when monitoring arrhythmias) regarding heart rate. Such devices could provide data for specific questions such as heart rate. Comparison of these devices with established methods such as 24 hour Holter monitoring would be important to document. It is not clear how this study could be used in such a situation. Authors need to further justify this study

This comment is similar to previous reviewers comments we have clarified our goals and added additional statements to introduction and limitations sections.

From an implementation component there are no striking errors in this manuscript. It should be noted however that the average heart rates of the dogs examined are actually low for atrial fibrillation. Dogs that have high ventricular response rates are of course the more critical for monitoring and this paper does not address that kind of patient. Differences between low and high ventricular response rate heart rates over 24 hours is likely meaningful.

We have added additional explanations for why our 24hour mean HR was considerably lower in this study to the limitations section.
