# Supplementary material for: Comparison of heart rate obtained from shorter duration Holter recordings to 24-hour mean heart rate in dogs with atrial fibrillation
Source: PLoS One. 2020 Nov 2;15(11):e0241620. doi: 10.1371/journal.pone.0241620 (PMC7605697; doi:10.1371/journal.pone.0241620)
Supplement: Attachment — Submitted filename: Response to Reveiwers.docx [file pone.0241620.s002.docx]

Reviewer #1: The authors have addressed most of my concerns. The only concern that I have is that individual dogs might have had "better agreement" than other dogs. A more complex analysis, where "dog" is inserted as a random variable into the model, might help demonstrate this.

To highlight this, one dog might have had agreement within 10% for 70% of the 5 minute segments, while another dog (for whatever reason) might have had agreement within 10% for only 10% of the 5 minute segments. A dog with an average AF rate of 230 bpm would have a higher probability of agreeing than one that still had considerable vagal influence on the AV nodal conduction.

This type of approach will likely require consultation with a statistician/epidemiologist. The authors might consider plotting the % agreement for each shorter period against average 24hr HR to see if there is any relationship.

We have now added an additional analysis that accounts for the random effect of dogs and added necessary discussions.

Reviewer #2: General comments: thank you for the opportunity to read this revised version of the manuscript – I would like to thank the authors for addressing the majority of my previous comments and concerns. I have some remaining questions, which are detailed below.

Abstract
Line 40-41 – you say in lines 37-38 that “nearly 100% of heart rate averages obtained from 2 hour ECG recordings fell within 20% range” – but here you says that all of them did – please clarify this inconsistency.

“Nearly 100%” was used instead of “all” in the lines 37-38 since the CI for the median included 100%. We have changed the wording for consistency.

Materials and Methods
Lines 158-159 – I’m still not sure why you are reporting both the range and the IQR. It seems superfluous to me – I would just report the range.

Removed all applicable IQR throughout the manuscript

Lines 161-163 – it is not clear from this whether you mean “within 10% of the mean” (i.e. mean +/- 5%) or “mean+/- 10%” (which is what it says in the abstract) – please reword this to improve clarity.

Recommended changes made to improve clarity.

Lines 161-163 – I assume that you mean that you are reporting the percentage of HR averages falling within 10%, 15% and 20% of the 24 hours mean HR FOR THAT DOG (rather than the average mean HR for all dogs)? Please clarify.

Clarification provided

Results
Line 174 – again, I don’t know why you are reporting both range and IQR.

Recommended changes made
Lines 198-200 – again, the “10% range” is not the same as “mean +/- 10%”; it is mean +/- 5%. Please make sure that the wording is clear throughout the manuscript.

Recommended changes made throughout the manuscript

Lines 198-201 – an additional way to look at this would be to report the percentage of measurements that would have resulted in a different clinical decision being taken. For example, if (for the purposes of the study) we take 125bpm as a cut-off for deciding whether or not to change medications, you could report what percentage of the shorter recording were above this cut off if the 24 hour mean was <125 (and vice versa). This would be easier for clinicians to interpret, I think.

We do see practical utility in providing clinical decision cut-off and accuracy for such a cutoff above and below. The accuracy of a binary cutoff would be naturally better than accuracy for a specific percent range from 24-hour mean HR. For example, for a dog with a 24 hour mean HR of 200bpm, the 20% range would include heart rates from 140 to 240 bpm. If for instance this dog’s minimum and maximum heart rate ranges from 130 – 220 using 1-hour averages at home, 100% of the values will be above 125 bpm while a lot lesser percentage of values will be between 180-220. However, the practical utility may be limited given that a clinician may not know the predicted 24-hour mean HR at the time of any short-term ECG evaluation. We have highlighted this information as well in the discussion. As such given the limited practical utility we have not added this information.

Table 1 – the poor accuracy for 3-hour recordings in the 10% range column is intriguing – the range includes 50% (i.e. no better than flipping a coin) – can you comment on the possible reasons for this apparent anomaly?

There are at least two scenarios. One such scenario would be a dog with a high 24-hour mean HR and if such dog can reach really low HR values at rest. In contrast also can happen in dogs with lower mean HR but has the ability to reach extremely high max HR during activity. In either case, the analysis looks for HR values within an acceptable percentage from the 24 hour mean HR. The probability of falling out of that range may be lower than a coin toss in some dogs. If it a cutoff such as value above and below 125 bpm was used, then the probability (similar to a coin toss) would be expected to be above 50%.

Discussion
Lines 232-234 – I appreciate what you are saying here (clearly, 20% of a larger number is bigger than 20% of a smaller number). However, I still think that even at lower heart rates 20% is too inaccurate – even at 120bpm, if you are talking about +/20% (which remains unclear – see previous comments) then this would go as high as 144bpm (which would almost certainly be interpreted differently). Even if you mean “within 20% of the mean” that would take you to 132bpm, which may well be interpreted differently from 120bpm. Personally, on the basis of your results I would want at least a 3 hour recording.

We agree that it is bit of a judgement call. We have now modified the sentence to better reflect what we are attempting to say.

Lines 247-250 – I sincerely hope that clinicians do not accept wider limits of accuracy! See previous comments.

That is our hope too.

Lines 251-257 – these two sentences appear to contradict one another – if the other at home tools have the capacity to record continuous ECG for more than one hour, why is it not feasible to use them to make recording >1 hour long? Should it not say “…do not have the capacity to record continuous ECG for more than one hour duration”?
Yes it should say do not have the capacity. We apologize for the oversight.

Conclusions
Lines 289-290 – that is a fair interpretation of your results – but I still maintain that the level of inaccuracy is potentially dangerous in terms of its impact on how the clinician might choose to manage the case (e.g. make dose adjustments to rate control medications (or not)). I think that it is very important that you acknowledge this overtly in the text – this is not simply a mathematical problem, but something that potentially impacts on how well patients are managed clinically.

We have added an additional statement to clarify this.

Lines 290-292 – I think that it is really important that you provide more information that helps the reader understand better what 20% inaccuracy actually means for their patients; otherwise how can they judge what an acceptable level of error is?

We have added an additional statement to provide clarification.
