# Supplementary material for: Comparison of heart rate obtained from shorter duration Holter recordings to 24-hour mean heart rate in dogs with atrial fibrillation
Source: PLoS One. 2020 Nov 2;15(11):e0241620. doi: 10.1371/journal.pone.0241620 (PMC7605697; doi:10.1371/journal.pone.0241620)
Supplement: Attachment — Submitted filename: Response to Reviewers.docx [file pone.0241620.s003.docx]

Lines 224-227 – surely the purpose of measuring the shorter duration heart rate averages is to try to predict the 24 hour mean heart rate; this analysis is therefore back-to-front.

This analysis was requested by reviewer one to highlight the individual differences between dogs in accuracy of short duration ECG.
Lines 227-229 – what is the P value for this effect?

As such there is no p values associated with this effect. It was calculated as a % of individual variances (random effect) to total residual variance. We have modified the wording to reflect it. Prior to selecting the model, models with and without dog as a random effect was compared and addition of random effect was found to be statistically significant.

Lines 229-232 – apologies if I have missed something, but I don’t understand where this information comes from – please can you explain how you derived this (i.e. where the information that “on…average 60% of the HR averages obtained from short duration ECG recordings fall within +/-10% of the 24 hour mean HR” comes from – I can’t figure it out from the preceding sentences).

The 60% in this sentence applies to the intercept of the linear mixed model.
Discussion
Lines 244-257 – I appreciate what you are trying to say – but this argument feels somewhat circular (essentially you can’t know what level of accuracy you need until you know what the 24 hour mean is, by which point you have done a 24h Holter, so the question becomes moot). Given this, can you suggest any situations in which a shorter recording WOULD be valuable? After all, surely the point of doing a 24h Holter in dogs with AF is that you don’t know what the 24h mean heart rate is and you want to find out? After all, even if you have previously Holtered a dog, surely the point of re-Holtering them is to see if anything has changed, so you don’t know what the mean HR is going to be before you do the Holter.

We appreciate your input on this. Prior to this publication, the accuracy of shorter duration ECG to predict 24 hour mean was unknown in AF dogs. If the shorter duration ECG predicted 24 hour mean HR better than the what was noted in this study, then certainly it could have replaced Holter monitoring for all dogs. But in this study the accuracy widely varied between dogs and was not influenced by the 24 hours mean HR, complicating further on selection of a desirable accuracy. We wanted to state this clearly to avoid any misleading information to the readers on the applicability of the results. As we pointed out in the discussion, one situation may be when the in-hospital ECG HR is above 155 bpm, then the 24-hour mean HR can be predicted to above 140 bpm. Then we would think 20% accuracy would be too high and a tighter accuracy is desired.

Conclusions
Lines 307-309 – I would still prefer this sentence to be edited or removed, as I don’t think that clinical functionality is determined by the clinician’s judgement (if something is wildly inaccurate that doesn’t change just because the clinician has decided that they are happy to accept a wildly inaccurate result).

This sentence is removed
Lines 309-314 – thank for adding this information – I think it provides very valuable context.
